# The EXPRESS Study: Exercise and Protein Effectiveness Supplementation Study supporting autonomy in community dwelling frail older people‐study protocol for a randomized controlled pilot and feasibility study

**DOI:** 10.1186/s40814-017-0156-5

**Published:** 2017-07-06

**Authors:** Agathe Daria Jadczak, Natalie Luscombe-Marsh, Penelope Taylor, Robert Barnard, Naresh Makwana, Renuka Visvanathan

**Affiliations:** 1 National Health and Medical Research Council Centre of Research Excellence Frailty and Healthy Ageing, University of Adelaide, Adelaide, South Australia Australia; 2 Adelaide Geriatrics Training and Research with Aged Care (G-TRAC) Centre, Discipline of Medicine, Adelaide Medical School, University of Adelaide, Adelaide, South Australia Australia; 3 Aged and Extended Care Services, The Queen Elizabeth Hospital, Central Adelaide Local Health Network, 28 Woodville Road, Adelaide, South Australia 5011 Australia; 4 Nutrition and Health Program, Health and Biosecurity Business Unit, Commonwealth Scientific Industrial Research Organisation (CSIRO), Adelaide, Australia; 5 Centre for Physical Activity in Ageing (CPAA), Central Adelaide Local Health Network, Adelaide, South Australia Australia

**Keywords:** Ageing, Frailty, Exercise intervention, Protein supplements

## Abstract

**Background:**

Research has repeatedly demonstrated that exercise has a positive impact on physical function and is beneficial in the treatment of physical frailty. However, an even more effective strategy for managing physical frailty might be the combination of multicomponent exercise with the intake of high-quality protein supplements, but the efficacy remains unclear for older adults who are already pre-frail and frail. The aim is to examine the feasibility of recruiting frail older adults to participate in a trial designed to determine the potential effects of a 6-month exercise and nutrition intervention on physical function. The feasibility objectives will include frail older peoples’ compliance, the safety and tolerability of the trial, the estimation of estimates to aid sample size calculation, and the potential efficacy. Primary outcomes for the main trial will include gait speed, grip strength and physical performance. Secondary outcomes will include frailty status, muscle mass, nutritional intake, physical activity levels, cognitive performance and quality of life.

**Methods/design:**

A randomised, parallel, control pilot and feasibility study will be conducted. All participants will be randomly assigned to either (a) an exercise program + high-quality protein supplement or (b) an exercise program + low-quality protein supplement. Both protein supplements will be matched closely in colour, flavour and packaging so that both the participants and the research staff are blinded. The exercise program will be the same in both groups. Assessments will be conducted at baseline and at 3 and 6 months and include gait speed, grip strength, the Short Physical Performance Battery, Timed Up and Go Test, FRAIL Screen, bioelectrical impedance analyses, 24-h dietary recall, Katz Activities of Daily Living, Lawton Instrumental Activities of Daily Living, the Trail Making Test, Short Form Health Survey-36, and 1 week accelerometer quantification. The data will be analysed using an ANCOVA model.

**Discussion:**

This study is expected to provide much needed insight into the feasibility of recruiting and retaining frail older adults into community-based intervention programs, while providing knowledge relating to the safety, tolerability and benefits of a combined exercise and protein supplement program designed to halt or reverse the transition of physical frailty in the community. If shown to be effective, this strategy could be included in the best practice clinical guidelines for community-dwelling older adults who are pre-frail or frail.

**Trial registration:**

Australian New Zealand Clinical Trials Registry, ACTRN12616000521426

## Background

Globally, the number of people aged 65 years and older is expected to triple over the next 30 years [1, 2]. In Australia, it is estimated that approximately 10 million people will be aged over 65 years by 2050 [3]. A common geriatric syndrome associated with ageing is frailty [4]. Frailty is defined as a “clinically recognisable state of increased vulnerability resulting from age-associated decline in reserve and function” [2] and results in increased morbidity, including disability, loss of independence, increased hospitalizations and reduced quality of life [5, 6]. While frailty is measured using different scales, one of the most commonly used instruments is the Fried Frailty Index [7], which defines frailty according to a physical phenotype consisting purely of physical components. This construct of a physical phenotype is regarded as being distinct from disability or co-morbidity, and hence, the Frailty Index is considered to be highly predictive of future decline in physical health.

Due to the appreciation of the Fried Frailty Index as a relatively easy tool with which to determine whether a person is physically frail, there have been a number of studies that have described the prevalence of this phenotype in different regions around the world. The Survey of Health, Aging and Retirement in Europe (SHARE) surveyed older adults across Europe and Israel using the Fried criteria [7], and the prevalence of frailty was reported to be 17% (range from 5.8% in Switzerland to 27.3% in Spain) [8]. Data from a longitudinal cohort of Australian women using similar frailty criteria reported the same prevalence of pre-frail and frail [9] and, by 2050, this is estimated to represent four million Australians [10]. These data provide impetus for the development of strategies that can be used to either halt or reverse the transition of physical frailty and the manifestation of poor health outcomes. Otherwise, increasing poor health and healthcare costs among this population cannot be mitigated [2, 11].

While physical frailty appears to be reversible when early intervention is provided [7, 9], there remains a lack of consensus regarding the management of older people who are frail or have complex health conditions. Evidence indicates that exercise is critical in managing many of the physiological changes that occur as individuals’ age [4, 12]. Exercise interventions, particularly those that include resistance training, have the potential to prevent, delay and reverse frailty [13]. For example, numerous studies have demonstrated that exercise maintains and restores one or more health parameters including muscle strength, bone integrity, balance and physical function in study populations that include older people who were relatively healthy (i.e. those without a clinical diagnosis) [14] and those classified as frail according to a variety of methods [12, 15]. However, the strength of the evidence from meta-analyses regarding the benefits of exercise for frail older people was limited by the fact that many of the studies reviewed included small and heterogeneous study populations. Few studies have simultaneously measured markers of muscle mass, strength and function; the type of control activity used as the comparator for an exercise intervention has been highly variable; and the compliance to programs has not been well reported. In addition, a study of 151 community-living pre-frail and frail older adults in Singapore showed that improvements in frailty status and a variety of biomarkers of physical frailty were largely comparable for the nutrition, physical, cognitive and combined treatments and all resulted in greater improvement compared with the control, i.e. standard care from health and aged care services in combination with a placebo nutritional supplement. However, while there were no statistically significant differences between the nutrition, physical, cognitive and combined treatments, the improvements in all measured domains where greatest with the physical and combined groups [16]. Accordingly, more studies are needed to identify feasible exercise programs, alone and in combination with other treatments, for community-dwelling pre-frail and frail older adults.

With respect to the nutritional management of pre-frail and frail older people, very few studies have been conducted to determine the effects of protein supplements, either on their own or in combination with exercise. Numerous studies have demonstrated substantial benefits of protein supplementation in combination with resistance exercise in healthy older adults [17, 18]. However, only three studies have been conducted in frail older adults and have yielded conflicting results for the benefits of protein on muscle mass and markers of strength and mobility [19–21]. One study reported increased mobility, but no increase in skeletal muscle mass or strength when consuming additional protein [19], while another showed that protein supplements do not increase the effects of high-intensity functional exercise [21]. Yet another concluded that resistance training, when combined with protein intake, did lead to increased muscle mass compared to resistance training alone [20]. Discrepant findings are likely to be the results of differences in study protocols and participants, with some study samples being too small, while others provided insufficient protein or administered the protein as a single bolus dose, or did not control for differences in background diet.

In addition to the aforementioned limitations of previous research conducted in the ageing field, another major issue are the varied tools used to identify pre-frail and frail older adults. There remains little agreement regarding the diagnostic test accuracy of any of the simple, common diagnostic tests for community-dwelling people. Although interest in the FRAIL Screen is increasing for a number of reasons, this quick, simple screening tool does not require measurements by health professionals and has been validated in various populations across the globe [22–24]. It includes five questions about fatigue, resistance, ambulation, illness and weight loss, representing the phenotypic definition for frailty [7]. The FRAIL Screen can be completed in 15 s. The brevity and simplicity make it useful as a screening tool for implementation in primary care, and it is said to be capable of detecting changes in frailty bidirectionally, and thus may be useful in monitoring for change [22].

Given the incompleteness of our knowledge relating to the efficacy of protein supplements in combination with exercise interventions on physical function in people aged 65 years and older and who are recognised as pre-frail/frail, more information is required. Without a fuller understanding of the effects of a supplement-exercise regimen on frail community-dwelling older adults, it will continue to be difficult, if not impossible, to successfully manage their condition.

## Methods/design

### Aim

The primary aim of this 6-month study is to report on the feasibility of recruiting frail adults aged 65 years and older using the FRAIL screen (e.g. source, speed) and retaining them for the entire 6-month intervention (e.g. compliance, safety, tolerability).

### Feasibility objectives

The feasibility objectives will include the recruitment of pre-frail and frail older people from the community using several recruitment channels, how well pre-frail and frail older people comply with the exercise and nutritional components of the intervention, their completion of the baseline, 3- and 6-month assessments and the safety and tolerability of the intervention. This information will be used to estimate the sample size required for the main trial and the potential efficacy of the main trial.

### Secondary patient-centred objectives

In addition, the study aims to determine the effects of exercise in combination with a protein supplement. The supplement will be either a (a) commercially available high-quality whey protein supplement or (b) a control supplement which is a commercially available lower quality rice protein. Changes in response to exercise in combination with either the low- or high-quality protein supplement will be determined by measurements of the following:

- Primary outcomes, including gait speed, grip strength and physical performance and
- Secondary outcomes, including frailty status, muscle mass, quality of life, nutritional intake, cognitive performance and physical activity levels

The primary and secondary outcomes listed above will potentially be used in the main trial after their feasibility is confirmed in this pilot trial.

### Study design

A randomised, parallel, control pilot study will be conducted in a community setting. Participants will be assessed at baseline and at 3 and at 6 months. Participants will be able to withdraw from the study at any time and will have their final assessments completed within 1 week of withdrawal.

### Participants

Participants will be eligible for this study if they are aged 65 years and older, able to converse in English, live in the community and are identified as being pre-frail (i.e. have a score ≥1 but <3 out of 5) or frail (i.e. have a score ≥3 out of 5) using the FRAIL Screen [22], including the following five questions:

- Are you fatigued?
- Do you have difficulties walking up one flight of steps?
- Are you unable to walk at least one block?
- Do you have more than five illnesses?
- Have you lost more than 5% of your weight in the past 6 months?

Participants with dementia (i.e. score 5 or less out of 10) as per the Rapid Cognitive Screen [25], severe renal impairment (eGFR ≤30 mmol/L) and those unable to comply with the exercise or nutrition study protocol will be excluded.

### Recruitment

**Fig. 1:**
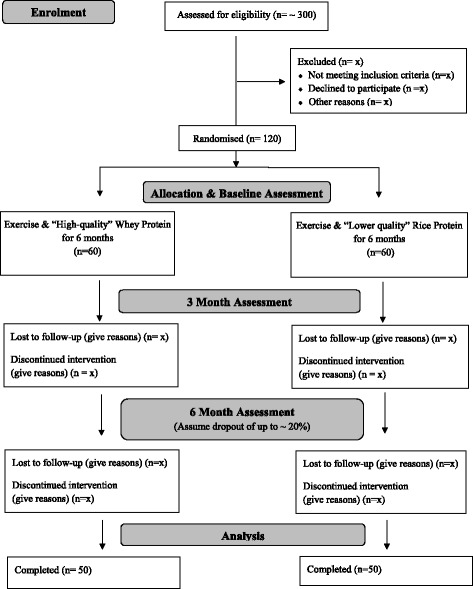
EXPRESS Study Consort Flow Diagram

Participants will be recruited through social media (e.g. Facebook), public seminars, local newspapers, radio and television advertising as well as flyers displayed in newsletters and the halls of collaborative aged care services, medical centres, GP practices and the Queen Elizabeth Hospital in Adelaide, South Australia, Australia. The flow of participants through the study will be captured as per the CONSORT statement and as depicted in Fig. 1.

### Randomisation and blinding

The study coordinator will enrol eligible participants into the study and send a participant’s identification number to the study biostatistician who will randomise the participants to one of the two study arms using a stratification system to ensure that an approximately equal numbers of men and women, aged <80 and >80 years, who are pre-frail (score ≥1 but <3 out of 5) and frail (score ≥3 out of 5), are allocated to each arm. The biostatistician will then notify the dietician on the allocated arm prior to the start of the intervention. The participants, as well as the study coordinator and research staff performing the study assessments, will be blinded to the two different types of protein supplements until the end of the trial.

### Exercise intervention

The exercise intervention will be undertaken by all participants who will be supervised by trained exercise physiologists. The exercise regimen includes one centre-based group exercise and two home-based exercise sessions, in addition to walking twice a week. During holiday periods or periods of illness, centre-based exercise can be missed, but participants will be asked to do an additional home-based exercise session if possible. These flexible approaches to the exercise regimen will be recorded.

The exercise prescription is based on the LIFE study, a physical activity intervention for community-dwelling frail older people conducted in the USA [13]. Multi-component exercises incorporating strength, balance, aerobics and flexibility were selected from the LIFE Study and modified for this pilot intervention. Strength exercises will be performed using own body weight, elastic exercise bands and ankle weights. The aerobic modality will be performed using walking exercise. The balance modality will include at least two balance tasks, and the flexibility modality will include the upper and lower extremities. Exercises that are safe and that can be executed in a sitting or safe standing position will be prescribed for the home-based exercise program. Additional exercises that may require more supervision will be included in the centre-based exercise sessions. The intensity of the exercises is based on the LIFE Study design using the Borg Scale [13, 26]. The Borg Scale allows a rating of perceived exertion (RPE) and can be used to monitor and control the intensity of exercise interventions. Strength exercises will be conducted at 15–16 RPE, and walking will be conducted at 12–14 RPE [26].

### Nutrition intervention

A qualified dietician will provide the nutrition prescription and instruct participants on how to prepare and consume the protein supplements and how to record them in the provided compliance diaries. All protein supplements will be provided as a powder in individual 26-g sachets to provide 20 g of protein. The protein powder (i.e. whey or rice protein) will be blended with a powdered flavour (i.e. vanilla) and reconstituted in ~150 ml of water by the participants at home. All drinks will be isocaloric (i.e. both supplying ~90 kcal per 26 g protein sachet) and isonitrogenous (~3.5 g of nitrogen per 26 g protein sachet) and of comparable taste, texture and aroma. The protein powder will be packaged in identical pharmaceutical grade silver foil sachets marked with an A or B to ensure that participants and the study coordinator and research staff performing all study assessments are blinded.

The protein load, source and timing of consumption are based on the recommendations by the PROT-AGE group [27] and the aforementioned meta-analyses [17, 18, 28]. Splitting the total load of protein into two supplements per day is based on evidence that a load of 20 g when combined with multicomponent exercise is sufficient to increase muscle protein synthesis in healthy, non-frail older people. During periods of hospitalisation, research staff will interact with the participant and treating health professionals to encourage, where safe to do so, continuation with the supplementation. Also, it is anticipated that participants will take sachets with them on holidays, thus continuing with supplementation.

### Screening and assessments

#### Screening

Participants will be screened for eligibility over the phone using the FRAIL Screen [22] and demographic- and health-related information will be collected. Medical clearance and serum creatinine investigation will then be obtained from the participants’ general practitioner before a home visit is scheduled for further screening. The home visit will include the Rapid Cognitive Screen (RCS) [25], the Charlson Co-morbidity Index [29], the Geriatric Depression Screen-5 (GDS-5) [30], Katz Activities of Daily Living (KATZ ADL) [31] and a safety check to ensure that participants exercise in a safe home environment. The screening process over the phone will take approximately 5 min and the home assessments will take approximately 30 min for each participant. The Short-Form Health Survey-36 (SF-36) [32] and the Lawton Instrumental Activities of Daily Living (Lawton iADL) [33, 34] questionnaire will be completed by the participants as self-assessments. At the completion of the screening, inclusion and exclusion criteria will be reviewed by the research team’s coordinator, and participants who fulfil all eligibility criteria will be enrolled into the 6-month nutrition and exercise intervention.

#### Completion of baseline, 3- and 6-month assessments

At baseline and at 3 and 6 months after commencing the nutrition and exercise intervention, assessments will be performed at the participants’ closest study centre (currently two locations including the Queen Elizabeth Hospital in the western suburbs and the Centre for Physical Activity in Ageing in the north-east of the city).

Baseline assessments will be conducted approximately 1 week after participants have completed the screening process and been enrolled into the study. These assessments will take approximately 1 h per participant and include the Trail Making Test [35], gait speed, grip strength (using a handgrip dynamometer) [36], Short Physical Performance Battery (SPPB) [37], Timed Up and Go Test (TUG) [38], bioelectrical impedance analyses (BIA) [39], 24 h dietary recall [40] and an accelerometer attached to the participant’s thigh [41]. The accelerometer will be collected 1 week later. At that time, participants will commence their exercise program (with the first session being a centre-based exercise class), consuming their allocated twice daily protein supplements.

The RCS [25], the Charlson Co-morbidity Index [29] and the Self Mini Nutritional Assessment (MNA-SF) [42, 43] will be conducted only at baseline to determine the participants’ risk of cognitive impairment, 5-year mortality and malnutrition.

A timeline of the screening process, interventions and assessments is displayed in Table 1. An overview of the assessment tools used to measure the potential primary and secondary outcomes related to the patient-centred objectives for the main trial at baseline and at 3 and 6 months is displayed in Table 2.

**Table 1:**
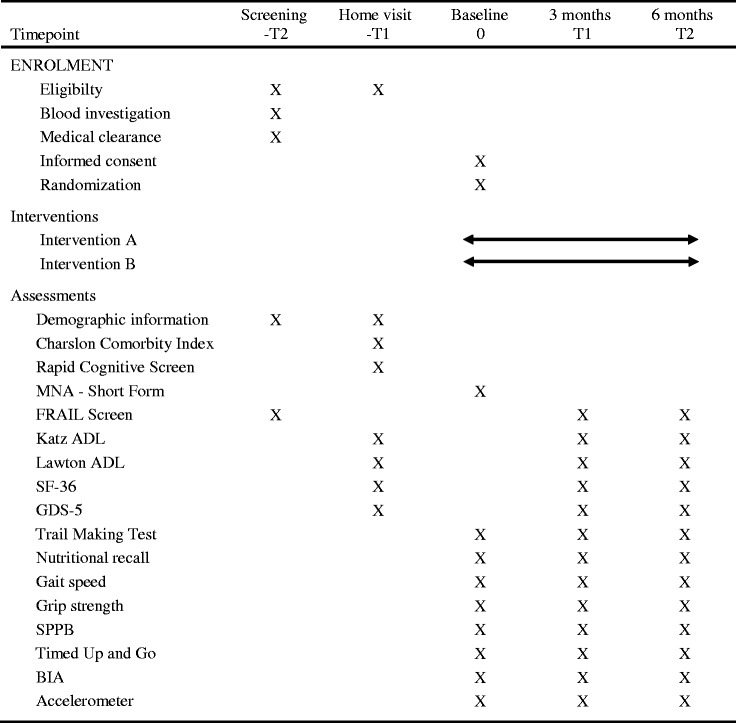
Schedule of enrolment, interventions and assessments

**Table 2:** EXPRESS Study assessments and cutoff criteria

EXPRESS Study assessments	Cut-off criteria		
Demographic information			
Charslon Comorbity Index	≤3 low risk	4–5 moderate risk	≥6 high risk
Rapid Cognitive Screen	8–10 normal	6–7 mild impaired	5 dementia
Mini Nutritional Assessment-Short Form	12–14 normal	8–11 at-risk	0–7 malnourished
FRAIL Screen	0 robust	1–2 pre-frail	≥3 frail
Katz Activitis of Daily Living	6 full function	4 moderately impaired	≤2 severely impaired
Lawton Instrumental Activities of Daily Living	0–3 high depended	4–6 moderate dependent	7–8 independent
Short-Form Health Survey-36			
Geriatric Depression Scale-5	≥2 answers: depression		
Trail Making Test	Part A: >78 s deficient	Part B: >273 s deficient	
Nutritional recall			
Gait speed	♂ 0.65/0.76 m/s (≤173/>173 cm)	♀ 0.65/0.76 m/s (≤159/>159 cm)	
Grip strength	♂ 29–32 kg according to BMI	♀17–21 kg according to BMI	
Short Performance Physical Battery	Limitation: 0–3 severe, 4–6 moderate, 7–9 mild, 10–12 minimal		
Timed Up and Go	<12 s normal mobility	≥12 s limited mobility	
Bioelectrical impedance analyses			
Accelerometer	Low activity ♂ <383 kcal/week	Low activity ♀ <270 kcal/week	

♂ male ♀ female

### Compliance and safety

Compliance diaries will be handed out to participants with instructions provided on how to complete them; specifically, they will be asked to record the timing and number of supplements consumed per day. The researchers will check compliance with the supplements and exercise prescription on a weekly basis for the first 2 weeks and then every 2 weeks thereafter if no problems are evident. If the study team identify that a participant’s compliance to the planned number of exercise sessions per fortnight, or the planned number of protein sachets, is less than 70% (i.e. <7 exercise sessions attended or <20 sachets consumed), the research exercise physiologist and dietitian will respectively interview the participant to assess any difficulties and identify strategies that could facilitate compliance.

For participants who have glomerular filtration rates (GFR) of >30 but ≤60 ml/min/1.73 m^2^, the GFR will be retested at 3 and 6 months, and if deterioration is noted (very unlikely), the medical registrar will liaise with the general practitioner and the participant for further assessment and management. Protein supplementation can be safely prescribed up to 1.2 g/kg body weight/day where GFR is >60 [27].

Adverse events (AEs), that is, any untoward medical occurrences (i.e. pain, discomfort, wind, headaches, noticeable difference in body and memory) will be investigated during all visits, whether or not they are related to the study product or physical regimen. The events will be reviewed by the study’s medical registrar and participants will be referred to the research physician as required.

### Statistical analyses

Statistical advice has been provided by a co-investigator (KL) who is a professional biostatistician; she will be responsible for overseeing all statistical analysis for the team. Descriptive analyses will be used to assess the completeness and variability of the outcomes using means, medians, standard deviations and ranges as appropriate. Mean and median outcomes within each group will be presented with 95% confidence intervals to help inform power calculations for a possible definitive trial. A preliminary assessment of the efficacy of the treatment will also be conducted via an intention-to-treat analysis. For each study outcome, the change from baseline after 3 and 6 months will be compared between the two study arms. These analyses will be estimated in an ANCOVA model with the 3- and 6-month value as outcome, and the baseline value age, gender and frailty status as covariates. The resulting treatment effect will be reported as the least square mean and 95% confidence interval for the primary outcomes of gait speed, grip strength and physical performance. A plot of changes over time for pre-frail and frail participants will be used to observe whether changes in the frailty status can be seen.

If recruitment is going to take substantially longer than 12 months, an interim analysis will be performed on the numbers of participants who have completed the study within that period, and a decision will be made as to whether the study is feasible to complete within a reasonable timeframe of an additional 12 months.

## Discussion

Frailty is a major public health issue. It directly, and also indirectly through increased disability, contributes to increased health and aged care costs of elderly populations around the world [2]. Frailty is a continuum of accumulated lifetime assaults on the body that compromises physical and/or mental equilibrium, predisposing older adults to increased dependency on healthcare resources [4]. Adults who are identified as being pre-frail have an increased risk over the next 3 to 4 years of becoming frail, requiring greater support in performing activities of daily living and maintaining independence [7]. This means the early detection of frailty is pivotal for timely interventions that may assist in managing this geriatric condition and reducing healthcare costs, and to date, there remains a paucity of information about lifestyle interventions that may be beneficial for this target population.

While exercise in combination with protein supplementation has been shown to positively impact a variety of physical and/or psychological health attributes in pre-frail and/or frail older people, several aspects related to the study design of previous research still limit the interpretation of the collective results. Accordingly, the proposed study protocol hopes to extend the current knowledge in this area by specifically examining the feasibility, tolerability, efficacy and safety of consuming twice daily protein supplements in the context of a multicomponent exercise program.

Recruiting and retaining frail older adults into community-based intervention programs is a major issue that prior research has identified and is generally the reason why many studies report relatively small numbers of participants (and especially small numbers of completers) [44]. Accordingly, this study has been designed to represent and test the efficacy of a pragmatic, community-led, exercise and nutrition program which is based on postulated hypotheses that remain to be debated within the fields of nutrition and gerontology [27]. Addressing many of the methodological limitations noted in the background literature, this study protocol should optimise the ability to recruit pre-frail and frail older participants who want to remain independent and engaged within their communities. For example, participants will be diagnosed as being pre-frail or frail using the FRAIL Screen which can be easily used in all primary care settings because it is simple and quick; as such, a variety of recruitment channels will target to promote the study including general practice clinics, age-care organisations, groups that are dedicated to supporting older adults and also the general public via social media channels. Secondly, on a weekly basis throughout the 6-month intervention, our multi-disciplinary research team consisting of geriatricians, age-care nurses, exercise physiologists and dietitians/nutritionists will be supporting each participant—albeit in a group setting—to build a study community that should assist with maintaining participant motivation. Thirdly, the personalised tailoring of the exercise program should also maximise the likelihood that each participant will progressively improve their strength, flexibility, balance and endurance, and thereby improve their physical health and overall wellbeing.

Regarding the protein supplementation part of the program, participants will be instructed to consume the daily protein supplements in close proximity (i.e. <1 h) to the completion of the exercise and in between meals on non-exercise days. The timing of consumption was based on the ongoing theory that resistance training increases amino acid delivery to the muscles, as well as absorption; hence, participants should be consuming an adequate amount of substrate, in a timeframe, that should maximise stimulation of muscle protein synthesis [45]. Moreover, there is consistent evidence that whey protein rather than casein, milk, soy or pea is more superior at promoting muscle protein synthesis or building muscle mass due to its higher quality (i.e. higher free amino acid to total amino acid content and especially its high essential branched-chain and leucine content) [46]. However, other plant-based proteins such as rice or pea protein are popular with consumers and while they are of lower protein quality, evidence regarding their impact on muscle mass, strength or physical performance/function, particularly when consumed over several months, remains scant. Therefore, this study will directly compare a whey protein supplement (i.e. higher quality protein) to a rice protein supplement (i.e. a lower quality protein) to address this concern. Long-term tolerability and safety to both proteins, as well as to the exercise program, will also be an important issue addressed within this study.

## Conclusion

This study is expected to provide much needed insight into the feasibility of recruiting and retaining frail older adults into community-based intervention programs, while providing knowledge relating to the safety, tolerability and benefits of a combined exercise and protein supplement program designed to halt or reverse the transition of physical frailty in the community. If shown to be effective, this strategy could be used to inform the design of cost-effectiveness trials which will be necessary if public health policy makers and funders are to be strategically influenced. Evaluation of the collective findings from current and future research will be critical to refine and extend the current best practice clinical guidelines for community-dwelling older people who are pre-frail or frail.

## Abbreviations

AE: Adverse event
BIA: Bioelectrical impedance analyses
CT: Computer tomography
GDS-5: Geriatric Depression Screen-5
GFR: Glomerular filtration rates
KATZ ADL: Katz Activities of Daily Living
Lawton iADL: Lawton Instrumental Activities of Daily Living
MNA-SF: Self Mini Nutritional Assessment
RCS: Rapid Cognitive Screen
RPE: Rating of perceived exertion
SF-36: Short Form Health Survey-36
SPPB: Short Physical Performance Battery
TUG: Timed Up and Go Test
